# Maternal and perinatal outcomes in urgent referral and non-referral cases of emergency cesarean section at a district hospital in Zambia: a retrospective observational study

**DOI:** 10.11604/pamj.2019.33.324.19832

**Published:** 2019-08-26

**Authors:** Yasuhiro Miyoshi

**Affiliations:** 1 Zimba Mission Hospital, Zimba, Zambia; 2 National Hospital Organization Nagasaki Medical Center, Nagasaki, Japan

**Keywords:** Cesarean section, urgent referral, indications, maternal outcomes, perinatal outcomes, Zambia

## To the editors of the Pan African Medical Journal

Cesarean section is the most commonly performed major surgical procedure in sub-Saharan Africa [1]. However, access to safe cesarean section is very poor. The cesarean section rate in Zambia is only 4.4% [2]. In rural Zambia, 42.0% of pregnant women deliver at home and 56.3% deliver at health facilities [3]. Many women deliver at the health centers nearest them, which lack doctors and advanced treatment capabilities. They must be transported to a district hospital if they need urgent cesarean section due to pregnancy and delivery complications. This study was undertaken to compare the maternal and perinatal outcomes and indications for emergency cesarean section between urgent referral and non-referral cases at a district hospital in Zambia. This retrospective observational study was conducted at Zimba Mission Hospital, Southern Province, Zambia. It accepts patients referred from approximately 10 health centers in the catchment area, which has a population of 98,000. In urgent cases of potential complication for mother or fetus, the nurses/midwives at health centers transfer the patients to the hospital by ambulance. Depending on the health center's location, this transport usually takes 1-6 h. Most of the roads are unpaved, and road conditions worsen in the rainy season. Most health centers do not have ambulances; the ambulance at the hospital typically must pick up the patients. Cesarean section can be started usually within 30 minutes to 2 hours after the decision depending on the various factors such as time, weather and availability of hospital vehicles to pick up the theater staff. All patients who underwent emergency cesarean section and all babies born by emergency cesarean section at Zimba Mission Hospital between 1^st^ January and 31^st^ December 2017 were included in this study. The indications for emergency cesarean section and maternal and perinatal outcomes were compared between urgent referral and non-referral cases. Data were analyzed using EZR (version 3.1.2; Saitama Medical Center, Jichi Medical University, Saitama, Japan). Statistical significance was defined as p < 0.05. This study was approved by the research ethics committees of the University of Zambia Biomedical Research Ethics Committee (No. 001-03-19) and the National Hospital Organization Nagasaki Medical Center (No. 30130).

Of the 1,704 deliveries occurring at the hospital in 2017, 1,400 (82.2%) were vaginal and 304 (17.8%) were by cesarean section. Most (n = 266, 87.2%) cesarean sections were performed emergently. The study sample comprised these 266 deliveries and the 277 resulting neonates. Ninety-two (34.6%) of these cases were referred urgently from other facilities, with the patients transported by ambulance, due to pregnancy and labor complications. The maternal, pregnancy, and delivery characteristics of the two groups were similar. The leading indication for emergency cesarean section was prolonged labor, followed by fetal distress. Compared with the referral group, the non-referral group contained more cases of prolonged labor (47.8% vs. 62.1%; p = 0.036) and fewer cases of preeclampsia/eclampsia (7.6% vs. 1.7%; p = 0.035) and uterine rupture (4.3% vs. 0%; p = 0.025). Maternal outcomes were similar in the two groups (Table 1). Apgar score <7 at 5 min was more common in the referral group than in the non-referral group (12.2% vs. 2.8%; p = 0.004). The perinatal mortality rate, including stillbirths and neonatal deaths, was higher in the referral group than in the non-referral group (13% vs. 1.1%; p < 0.001; Table 1). Our findings reflect the delays of cesarean section in referral cases. The situation can be characterized using a “three-delay” framework-first, the delay in deciding to seek care by the mother and/or her family; second, the delay in reaching the health center or hospital once the decision to seek care is made; and third, the delay in receiving adequate care once at the facility [4]. To reduce the first delay, the education of pregnant women is important. They must be encouraged to attend antenatal visits at health centers to enable health care providers to address high-risk pregnancies requiring hospital delivery. Reducing the second delay is also important. Longer travel times from health centers to district hospitals are associated with adverse neonatal outcomes [5]. The reduction of transport delay through improvement of the referral system is important. Each health center should have its own ambulance, and road conditions should be improved. To reduce the third delay, the education of nurses/midwives at the health centers is the main measure. These nurses/midwives need to assess patients' risks and refer them to the hospital at appropriate times. The efforts should be made to reduce the time gap between the time of decision for cesarean section and the incision at the hospital.

**Table 1:** Maternal and perinatal outcomes

Maternal outcomes			
Characteristics	Non-referral (n = 174)	Referral (n = 92)	P-value
Estimated blood loss	737 ± 420^a^	871 ± 826^b^	0.662
Postpartum hemorrhage	23 (13.2)	16 (17.4)	0.537
Bladder injury	1 (0.6)	2 (2.2)	0.572
Wound infection	5 (2.9)	1 (1.1)	0.668
Maternal mortality	2 (1.1)	0 (0)	0.77
**Perinatal outcomes**			
**Characteristics**	**Non-referral (n = 179)**	**Referral (n = 98)**	**P-value**
Male	100 (57.8)^c^	61 (62.2)	0.557
Mean birth weight	3,201 ± 506^d^	3,079 ± 632	0.41
Apgar score <7 at 5 min	5 (2.8)	12 (12.2)	0.004
Perinatal mortality	2 (1.1)	13 (13.3)	< 0.001
Stillbirth	2 (1.1)	7 (7.1)	0.019
Neonatal death	0 (0)	6 (6.1)	0.004

a 24 missing data

b 12 missing data

c 6 missing data

d 7 missing data

Values are given as mean ± standard deviation and number (percentage)

## Conclusion

The referral group had more severe indications for and poorer perinatal outcomes of cesarean section than did the non-referral group. Poor outcomes could be related to the delays of cesarean section. The efforts should be made to reduce the delays.

## Competing interests

The author declares no competing interests.
